# Paramyxovirus Entry and Targeted Vectors for Cancer Therapy

**DOI:** 10.1371/journal.ppat.1000973

**Published:** 2010-06-24

**Authors:** Roberto Cattaneo

**Affiliations:** Department of Molecular Medicine, Mayo Clinic, and Virology and Gene Therapy track, Mayo Graduate School, Rochester, Minnesota, United States of America; University of California San Francisco, United States of America

## Certain Viruses Cause Cancer, but Reprogrammed Ones May Cure It

About a century ago, shortly after viruses were recognized, occasional tumor regressions were documented after natural infections. This observation established the idea of using viruses to fight cancer [1]. Early virotherapy clinical trials based on natural viruses were poorly controlled, but recent ones based on modified viruses are subject to extensive monitoring of viral replication, gene expression, and host immunity. Therapeutic efficacy is being assessed by well-defined biological end points, and can be improved [2]. For future clinical trials, more specific and potent oncolytic viruses are being developed based on three principles: targeting, shielding, and arming [3].

Box 1 lists several strategies currently utilized for each category of modification; not all modifications are applicable to all viruses, but interesting combinations of modifications can be applied to many viruses to enhance therapy, as recently discussed [3]. We focus here on the contribution of paramyxoviruses to the development of the next generation of cancer therapeutics, and in particular on targeting viral entry to cancer cells (Box 1, top two lines). We also bring examples of how paramyxovirus envelopes can shield oncolytic viruses from pre-existing antibodies, as well as target viruses of other families, in particular retro- and lentiviruses. Finally, we present one example of arming that enhances efficacy of virotherapy through its direct integration into a chemotherapy regimen, locally amplifying its effect. We note that modern virotherapy, while based on the creative application of basic knowledge derived from the study of viruses, is driven by the need for new alternatives for cancer treatment. Thus, while work creating and validating the next generation of vectors progresses, current clinical trials are based on vectors developed 5–10 years ago [4].

**Box 1. Virus Reprogramming: Three Principles, Many Combinatorial Options**

**TARGETING**

- **Entry – I: receptors**
- **Entry – II: particle activation** (proteases)
- Post-entry – I: transcription and replication (promoters)
- Post-entry – II: selective replication (cancer cell defects)

**SHIELDING**

- **Envelope exchange** (serotypes, or related viruses)
- Chemical (polymer coating)
- Biological (infected cell carriers)
- Local treatment (intratumoral application)
- Temporary immunosuppression (cyclophosphamide)

**ARMING**

- **Prodrug convertases** (e.g., PNP/fludarabine)
- Iodine symporter
- Pro-apoptotic proteins
- Selective disarming in normal cells (interferons, GM-CSF)

(Examples discussed in the text are bolded.)

## Targeting I: Paramyxoviruses Can Enter Cells through Designated Receptors

Not all viruses can be readily targeted to enter cells through designated receptors. Cell entry targeting of icosahedral viruses like adenovirus is complicated by the multiple constraints of their capsid symmetry: maintaining efficient assembly while modifying the capsid proteins is a challenge, but some success has been reported based on the display of short peptides [5]. On the other hand, targeting of enveloped, non-icosahedral viruses has progressed more rapidly utilizing large specificity domains, including single chain antibodies, displayed on the envelope proteins.

From the beginning, the envelope of paramyxoviruses was considered an attractive targeting substrate because receptor attachment and fusion function are separated on two proteins. In contrast, a single protein in retroviruses performs both functions, which has complicated retargeting strategies. The two-protein entry system of paramyxoviruses is also simpler than those of large DNA viruses that use several proteins. Among paramyxoviruses, which are non-segmented negative-strand RNA viruses with a helical capsid, targeting of the measles virus (MV) envelope is the most advanced.

**Figure 1:**
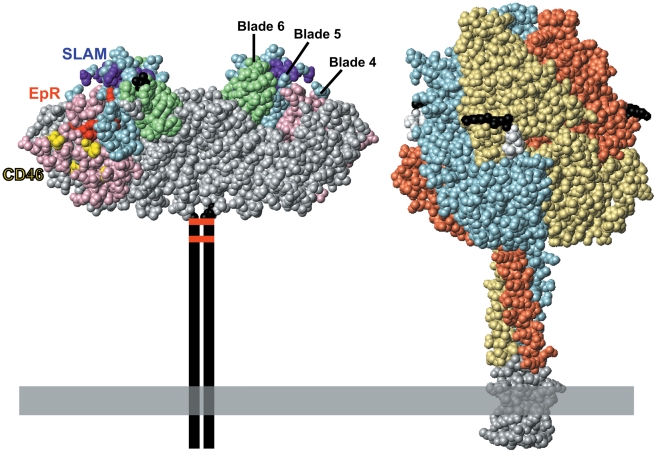
The paramyxovirus envelope glycoproteins. **Left**, space-filling representation of the crystal structure of the MV H-protein dimeric head [24]. The H-protein head has a beta-propeller structure with six blades; beta-propeller blades 4, 5, and 6 are surface shaded pink, blue, and green, respectively. SLAM-, CD46-, and EpR-specific residues are shaded purple, yellow, and red, respectively. The first and the last amino acid in the crystal structure (residues 154 and 607, respectively) are shown in black. The stalk, transmembrane region, and cytoplasmic tail of the H-protein are represented by two vertical lines. The two disulfide bonds that hold the H dimer together are represented by horizontal red bars. **Right**, space-filling representation of the crystal structure of the F-protein trimer of the paramyxovirus PIV5 [25]. Sequence conservation suggests that this structure is similar to that of the MV F-protein, for which there is no crystal structure. The three monomers are shown with different colors for clarity. The five residues preceding the cleavage site are shown in black, the five following it in white. Two arginines preceding the cleavage site were deleted to avoid proteolytic cleavage and support crystallization. A trimeric coiled-coiled domain appended to the F-protein ectodomain to mimic the transmembrane domain is shown in grey. The membrane is illustrated as a horizontal grey box.

MV enters cells by envelope–membrane fusion at the cell surface at neutral pH. Two glycoproteins mediate this process: the hemagglutinin (H) and fusion (F) proteins (Figure 1). The H-protein binds to receptors, while the F-protein mediates fusion of the viral and cellular membranes. H naturally interacts with at least three different receptors. The wild-type virus primarily uses the signaling lymphocyte activation molecule (SLAM, CD150) expressed on certain immune cells, while the vaccine strain has gained the ability to also use the ubiquitous membrane cofactor protein (MCP, CD46), a regulator of complement activation. Additionally, MV infects airway and bladder epithelia through an as yet unidentified receptor (epithelial cell receptor, EpR). The footprints of the three receptors on H have been characterized (Figure 1) [6], and it was shown a decade ago that MV attachment and cell entry can be readily targeted to designated receptors by adding specificity determinants to the H-protein carboxyl-terminus [7]. It was then demonstrated that many larger specificity determinants, including single chain antibodies, can be used to target MV entry [6]. These specificity determinants are connected to the H-protein through a flexible linker and are likely displayed on top of the H-protein head, as F and H have tight lateral interactions.

For cancer treatment, specific receptors were chosen among the targets of approved cancer therapeutics: for example, the lymphoma therapeutic antibody Rituximab targets the B-lymphocyte marker CD20. Thus, a CD20-targeted MV was generated and shown to prolong survival of immunodeficient mice in a lymphoma model based on CD20-expressing B-cell xenografts [8]. What was initially missing was de-targeting from the natural receptors CD46 and SLAM, but once the key residues of MV H supporting entry through these receptors were mapped and mutated [9], MV with fully retargeted entry were produced and their efficacy confirmed in pre-clinical trials [10]. The single chain antibody-based MV targeting system is versatile: many retargeted MV have been generated and shown to be effective in different animal models of oncolysis [3].

## Targeting II: Paramyxovirus Entry Can Be Activated through Cancer-Specific Proteases

In situ activation through cancer-specific proteases is a second targeting layer that can be applied to paramyxoviral envelopes. This concept is based on the modification of the F-protein, which requires protease cleavage for activation, and was developed for both Sendai virus and MV [11], [12]. Cleavage of the respective F-proteins was made dependent on a matrix metalloprotease, MMP-2, which recognizes and cleaves a specific hexapeptide sequence. MMPs are zinc-dependent endopeptidases that promote tumor progression by cleaving the extracellular matrix, and are up-regulated in almost every type of human cancer [13]. A recombinant virus (MV-MMP) was generated with a variant of the hexapeptide recognized by MMP-2 appropriately engineered into the F-protein, and was unable to propagate or produce a cytopathic effect unless it was added to cells expressing MMP-2. In mice, MV-MMP retained full oncolytic activity when inoculated into MMP-positive subcutaneous cancers, but unlike wild-type MV, MV-MMP did not infect and kill susceptible mice after intracranial inoculation, illustrating the enhanced safety of the virus [12].

This retargeting strategy, based either on MMP or other proteases [14], can be adapted to restrict cellular entry of other enveloped viruses that have protease-activated F-proteins. Viruses with such proteins currently in cancer clinical trials include herpes simplex (HSV) among the enveloped DNA viruses, and Newcastle disease virus among the paramyxoviruses.

## Fitting Targeted Envelopes on Capsids of Other Viruses; Shielding MV-Based Oncolytics

Entry retargeting of other enveloped viruses, especially retro- and lentiviral vectors, has proven difficult. To address this challenge, MV glycoproteins were incorporated into lentiviral vectors [15]. To sustain efficient incorporation of the MV glycoproteins in these vectors, it was necessary to precisely trim their respective cytoplasmic tails. HIV nucleocapsids pseudotyped with the CD20-targeted MV glycoproteins could deliver a reporter gene with great specificity and efficiency to CD20-expressing primary human lymphocytes. This work is important because it proves that the versatile MV envelope-based targeting system can be transferred to the most advanced vectors used for correction of genetic diseases [16].

While MV glycoprotein-retargeted lentiviral vectors have the potential to reduce off-target integration of vector genomes in trials based on ex vivo gene transfer, the prevalence of MV neutralizing antibodies in human sera interferes with the systemic delivery of these vectors. To circumvent neutralization issues, the use of envelopes from non-human paramyxoviruses have been considered. In particular, the envelope glycoproteins of Tupaia paramyxovirus [17] and canine distemper virus, which can be retargeted with single chain antibodies, have minimal if any cross-reactivity with human sera (K. C. Yaiw, J. Lampe, G. Ungerechts, R. Cattaneo, unpublished data). Once these retargeted paramyxoviral envelopes are properly fitted onto lentiviral vectors, such vectors could be inoculated systemically for targeted gene transfer.

This shielding principle can also be applied to MV vectors: the envelopes of non-human paramyxoviruses can be fitted onto MV nucleocapsids to produce chimeric viruses that evade pre-existing MV immunity, at least temporarily. We refer to a recent review [3] for discussion of this and other shielding principles that are currently being tested in pre-clinical and clinical trials of oncolytic viruses (see also Box 1, shielding). In short, polymers have been used to shield icosahedral viruses, in particular adenovirus, while cell carrier shielding has been used for many viruses, including MV. It is also revealing that several current clinical trials focus on cancer types that can be treated locally, like glioma in the brain or ovarian carcinoma in the peritoneum, avoiding intravenous injections and immediate neutralization. Finally, host immunosuppression with cyclophosphamide prior to virus administration enhances oncolytic efficacy, likely by suppressing host innate and adaptive immunity and temporarily favoring virus replication.

## Arming: Reprogrammed Viruses Enhancing Established Cancer Therapeutics

A fundamental paradigm of cancer therapy is that no single drug or treatment will cure cancer. Therefore, most therapeutic regimens for cancer are based on combinations of drugs, radiation, and surgery to maximize patient survival. As oncolytic viruses have so far provided incomplete cancer cures, the field has moved towards combining these viruses with traditional therapies. The most promising new avenue of experimentation is to integrate different components of current cancer therapy regimens with reprogrammed viruses expressing specific transgenes [18].

One example of this integrative approach is based on an armed and targeted MV that may improve the efficacy of the FCR (fludarabine, cyclophosphamide, Rituxan) regimen. This regimen is the front-line treatment for select non-Hodgkin lymphoma, and is based on cycles of treatment with fludarabine phosphate, cyclophosphamide/cytoxan, and the anti-CD20 antibody Rituxan. As an alternative to Rituxan, a CD20-targeted MV was considered. This vector was also armed with the prodrug convertase purine nucleotide phosphorylase, which converts fludarabine phosphate to a highly diffusible substance that is capable of efficiently killing bystander cells. The CD20-targeted and convertase-armed MV was shown to synergize with fludarabine to achieve oncolytic efficacy after systemic inoculation in a mantle cell lymphoma xenograft model [19]. Recently, precise timing of cyclophosphamide, virus, and fludarabine administration was shown to increase the window of therapeutic opportunity [20]. An alternative approach foresees arming CD20-targeted MV with the thyroidal natrium iodide symporter (NIS) gene, enhancing bystander effect by exploiting infected tumor cells to efficiently and locally concentrate radioiodine [21]. Again, this strategy improves on current clinical trials of non-Hodgkin lymphoma based on the I-131 labeled Tositumomab monoclonal antibody.

In conclusion, the first oncolytic virus approved as a cancer drug has been administered to thousand of patients in China [22], multiple viruses with improved oncolytic properties are currently being tested in well-controlled clinical trials, and the next generation of targeted viruses capable of integrating chemo- and radiotherapies is approaching clinical testing. Virus-based cancer therapies are on the horizon and rapidly approaching [23].
